# Physical Activity and/or High Protein Intake Maintains Fat-Free Mass in Older People with Mild Disability; the Fukuoka Island City Study: A Cross-Sectional Study

**DOI:** 10.3390/nu11112595

**Published:** 2019-10-29

**Authors:** Rie Takae, Yoichi Hatamoto, Jun Yasukata, Yujiro Kose, Takaaki Komiyama, Masahiro Ikenaga, Eiichi Yoshimura, Yosuke Yamada, Naoyuki Ebine, Yasuki Higaki, Hiroaki Tanaka

**Affiliations:** 1 Graduate School of Sports and Health Science, Fukuoka University, 8-19-1 Nanakuma, Jonan-ku, Fukuoka 814-0180, Japan; lrietty.tl@gmail.com (R.T.); mt.komi51@gmail.com (T.K.); 2 Institute for Physical Activity, Fukuoka University, 8-19-1 Nanakuma, Jonan-ku, Fukuoka 814-0180, Japan; yhatamoto@nibiohn.go.jp (Y.H.); yasujun0326@yahoo.co.jp (J.Y.); yujiros.day.717@gmail.com (Y.K.); m.ikenaga82@gmail.com (M.I.); hirotana47@gmail.com (H.T.); 3 Department of Nutrition and Metabolism, National Institute of Health and Nutrition, National Institutes of Biomedical Innovation, Health, and Nutrition, 1-23-1 Toyama, Shinjuku-ku, Tokyo 162-8636, Japan; yyamada831@gmail.com; 4 Faculty of Sports and Health Science, Fukuoka University, Fukuoka, 8-19-1 Nanakuma Jonan-ku, Fukuoka-shi, Fukuoka 814-0180, Japan; 5 Center for Education in Liberal Arts and Sciences, Osaka University, 1-17 Machikaneyamachou, Toyonaka, Osaka 560-0043, Japan; 6 Department of Food and Health Sciences, Prefectural University of Kumamoto Faculty of Environmental and Symbiotic Sciences, 3-1-100 Tsukide, Higashi-ku, Kumamoto 862-8502, Japan; eyoshi@pu-kumamoto.ac.jp; 7 Faculty of Health and Sports Science, Doshisha University, 1-3 Miyakodani Tatara, Kyotanabe-shi, Kyoto 610-0394, Japan; nebine@mail.doshisha.ac.jp

**Keywords:** physical activity level, body composition, protein intake, doubly labelled water, mild disability

## Abstract

Body composition changes with age, with fat mass (FM) increasing and fat-free mass (FFM) decreasing. Higher physical activity and high or adequate protein intake are thought to be beneficial in preventing the loss of skeletal muscle mass in the elderly. We aimed to investigate the relationships between physical activity, protein intake, and FFM in older people with mild disability. Total energy expenditure (TEE) under free-living conditions was assessed using the doubly-labelled water (DLW) method, and physical activity was measured using a triaxial accelerometer. Dietary intake was assessed using a self-recorded food intake diary during the DLW period. Percent FFM was significantly positively correlated with protein intake and physical activity level (PAL) after adjustment for age and sex (protein intake r = 0.652, *p* < 0.001, PAL r = 0.345, *p* = 0.011). In multiple linear regression analysis, when PAL, moderate-to-vigorous physical activity (MVPA), or protein intake were included, 31%, 32%, and 55%, respectively, of the variation in %FFM was explained. Moreover, the addition of both PAL/MVPA and protein intake explained 61%/60%, respectively, of the variation in %FFM. Either protein intake above the currently recommended level or higher levels of physical activity would be beneficial for the maintenance of high %FFM.

## 1. Introduction

Body composition changes with age, with fat mass (FM) increasing and fat-free mass (FFM) decreasing [1]. This age-related loss of muscle mass, strength, and quality, referred to as sarcopenia is associated with a higher risk of disability [2] and all-cause mortality [3]. Consequently, a reduction in the age-related loss of muscle mass may ameliorate the symptoms of sarcopenia, frailty, and other age-related disease processes. Chronic diseases are one of the common precursors for inducing disability [4]. Also, high body mass index (BMI) increase levels of frailty, suggesting body composition would be taken account for disability [5].

The encouragement of high levels of physical activity may be a useful strategy for the prevention of muscle loss during aging. Indeed, a systematic review has shown that it has the potential to prevent or reduce the progression of sarcopenia in the elderly [6], and high physical activity levels partially prevent the age-associated decrease in fiber cross-sectional area [7]. A decline in physical activity with aging may be the factor responsible for the reductions in skeletal muscle mass, strength, and quality observed. In which case, higher physical activity in the elderly would likely be beneficial for the prevention of muscle loss.

Another potential strategy for the prevention of muscle loss is the maintenance of adequate protein intake, because the anabolic response of skeletal muscle to dietary protein ingestion is lower in older people [8,9]. The recommended dietary allowance (RDA) for protein is 0.8 g/kg/day in the USA [10] and 0.85 g/kg/day in Japan [11], and this has been thought to be sufficient to maintain muscle mass and prevent muscle loss. However, in a recent study, a higher intake was shown to be beneficial and free of side effects [12]. In addition, another previous study has shown that lower protein intake may be a modifiable risk factor for mobility limitation [13]. Therefore, it is not clear whether older people consuming adequate or larger amounts of protein have higher muscle mass than those consuming less.

Taking the results of these previous studies together, either adequate protein intake or a high level of physical activity are likely to be effective in maintaining muscle mass in older people [14]. Previous review reported that additional protein supplementation did not seem to have a significantly greater effect on the exercise-induced increase in fiber cross-sectional area in the older group, despite the fact that the older protein-supplemented group showed a greater increase in muscle strength [15]. Recent intervention studies have shown that higher protein intake in combination with greater physical activity increases fat-free mass (FFM) in the elderly [16]. We therefore need to know older people who undertake a high level of physical activity and consume adequate dietary protein may be able to maintain higher muscle mass than those who do not. However, few studies have evaluated the relationship of both protein intake and physical activity with muscle mass in an older population under free-living conditions [17].

The purpose of this cross-sectional study was to investigate the relationships between physical activity level (PAL), protein intake, and FFM, and to determine whether high PAL and protein intake might result in higher FFM in community-dwelling older people with mild disability. We hypothesized that individuals who both undertake higher levels of physical activity and consume larger amounts of protein would have higher FFM than those who undertake low levels of physical activity and/or consume less protein, under free-living conditions.

## 2. Materials and Methods

### 2.1. Participants

**Figure 1:**
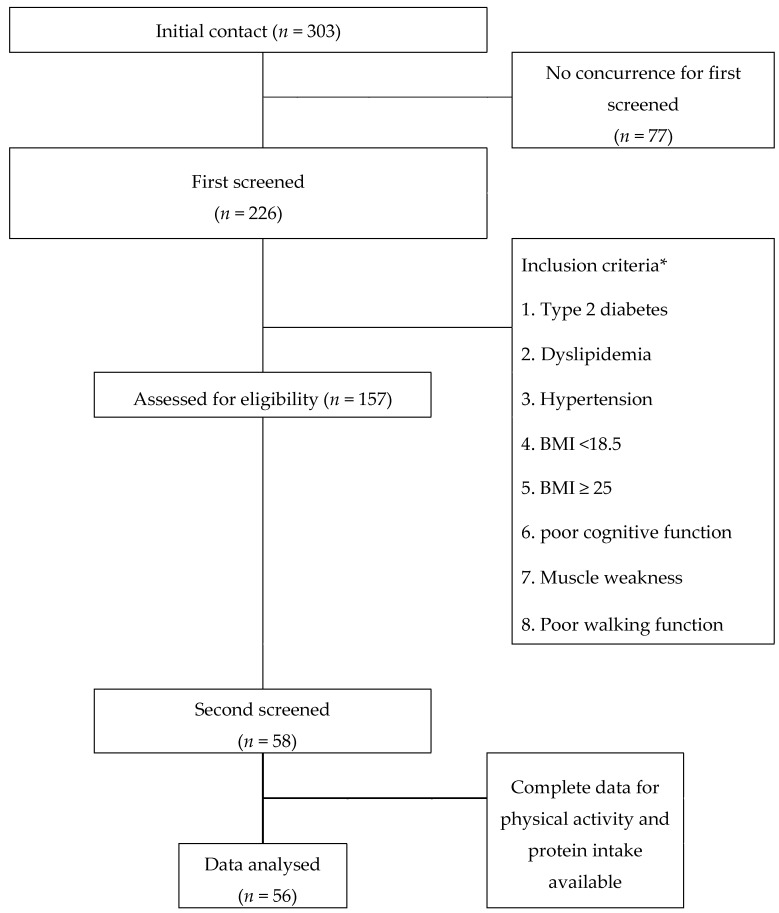
Flow diagram for the identification, screening, eligibility and statistical analysis conducted in this study. *: The detailed criteria are shown in the Methods section. BMI: body mass index.

The definition of mild disability was either (1) Type 2 diabetes, (2) Dyslipidemia, (3) Hypertension, (4) BMI < 18.5, (5) BMI ≥ 25, (6) poor cognitive function, (7) Muscle weakness, (8) Poor walking function. Participants were recruited through 18,000 flyers distributed in Higashi-ku. Three-hundred-and-three people (55–89 years of age) responded to the flyers, and participated in the first screening. For the first screening, 226 participants were evaluated for cognitive function, physical function, height, and weight. They also answered about the presence of the lifestyle-related disease in the questionnaire, such as type 2 diabetes, dyslipidemia (fasting serum triglycerides over 150 mg/dL, serum LDL-cholesterol over 120 mg/dL or HDL-cholesterol under 40 mg/dL), or hypertension (resting blood pressure [BP] over 150 mmHg systolic and/or 90 mmHg diastolic). Cognitive function was evaluated using a simple screening test that was developed with reference to the Hasegawa Dementia Scale [18], consisting of four test tasks involving an immediate memory test, temporal orientation test, three-dimensional visual-spatial perception test, and delayed recall test [19]. The total score was 15 points with a cut-off point of 12. Poor cognitive function is under 12 points [20]. Physical function was evaluated using a grasping power and walking speed that muscle weakness and/or poor walking function defined walking speed under 1 m/seconds and/or grasping power man: Under 25 kg, woman: Under 20 kg [21]. Secondly, one-hundred-and-fifty-seven participants were selected according to the above eligibility criteria. After receiving informed consent, 56 older people resided for analyses (Figure 1). Participants’ characteristics are described in Table 1.

This study was conducted according to guidelines laid down in the Declaration of Helsinki, and all procedures involving human participants were approved by the Ethics Committee of Fukuoka University in Japan (approval no. 15-04-02). Written informed consent was obtained from all participants. This study was registered under the title ‘Longitudinal Study of the Prevention of Dementia and Sarcopenia by Physical Assessment and Intervention in the Community-dwelling older adults: the Fukuoka Island City Study’ (registration no. UNIM-CTR UMIN 000036659; URL: https://upload.umin.ac.jp/cgi-open-bin/ctr_e/ctr_view.cgi?recptno=R000041766).

### 2.2. Anthropometric and Body Composition Measurements

All anthropometric measurements were performed with the participants in bare feet and wearing light clothes. BMI (body weight (kg)/height (m)^2^ was calculated from height and body weight measurements made using a wall-mounted stadiometer (3× million 50 m, YAMAYO MEASURING TOOLS Co., Ltd., Tokyo, Japan) and electronic scales (Innerscan BC-509, TANITA Co., Tokyo, Japan), which were recorded to the nearest 0.1 cm and 0.1 kg, respectively.

Body composition was calculated from the body water content, obtained using the stable isotope dilution method. This method involves loading the body with water labelled with stable isotopes of hydrogen (^2^H) and oxygen (^18^O), and it permits the measurement of total body water (TBW) using the dilution principle. FM and FFM were then calculated from the TBW: FFM was obtained by dividing the calculated TBW by the hydration coefficient of 0.732 for adults [22]; FM was then calculated by subtracting the derived FFM from the body weight.

### 2.3. Energy Expenditure and PA

We assessed physical activity using a triaxial accelerometer and calculated PAL from total energy expenditure (TEE), estimated using the doubly-labelled water (DLW) method. Triaxial accelerometer provides an accurate and detailed assessment of physical activity using the three axes of the inclinometer output and vector magnitude data [23], and TEE measured using the DLW method is acknowledged to be the “gold standard” method for the measurement of energy expenditure under free-living conditions [24,25]. The methods of assessment of physical activity are capable of indicating which physical activity parameter under free-living conditions is most important for the maintenance of FFM in community-dwelling older people with mild disability.

TEE was measured for 16 days using the DLW method. A urine sample was acquired for the measurement of baseline ^2^H and ^18^O enrichment. Each participant was given a drink containing a premixed dose of approximately 0.12 g/kg estimated TBW of ^2^H_2_O (99.8 at.%, Taiyo Nippon Sanso, Tokyo, Japan) and 2.5 g/kg estimated TBW of H_2_^18^O (10.0 at.%, Taiyo Nippon Sanso) [26]. The isotopically-labelled water equilibrates with the body water over several hours, after which urine samples are collected and analyzed to determine the elimination rate. A standard procedure was adopted that included the collection of four urine samples: On day 1 following the DLW administration, then on days 2, 15, and 16. Each urine sample was analyzed by isotope ratio mass spectrometry using automated analyzers of ^2^H and ^18^O (Hydra 20-20, Sercon Ltd., Crewe, UK). The difference between the elimination rates of the two isotopes is proportional to carbon dioxide production, which is used to calculate TEE.

The intensity of PA was quantitatively assessed using a triaxial accelerometer (Actimarker EW4800, Panasonic Electric Works Co., Ltd., Osaka, Japan) that was attached to an elastic belt and worn at the back of the waist for the entire 2-week period [23]. Participants were instructed to maintain their normal PA during the study. All participants were asked to wear their accelerometers 24 h a day for 16 days, exclusive of any time spent bathing or in water. The first and last days and any days on which ≤300 steps were counted or there were ≤10 min of over 2 × metabolic equivalents (METs) per day were excluded from analyses [27,28]. Activity was defined as one of five intensity levels. Using these data, the time spent in sedentary activities (1.1 to <1.5 METs), light (1.5 to <3.0 METs), and MVPA (≥3.0 METs) intensity PA was determined and expressed as the total daily duration of each (e.g., min/week or h/day) [23,29,30]. For inclusion in the analysis, the participants needed a minimum of 4 days with a minimum of 10 hr/day wearing time [31]. Participants excluded, if less than above criteria.

PAL was obtained by dividing the calculated TEE by the rest metabolic rate (RMR). RMR was estimated using the Ganpule equations for adult men and women. Ganpule equations use body size and body composition and are useful for estimating metabolic rates in the Japanese population [32].

### 2.4. Dietary Assessment

The dietary intake of nutrients was estimated using both self-reporting methods and visual records using a digital camera or mobile phone over 3 days (2 weekdays and 1 day at the weekend) during the DLW [33,34]. The participants were then interviewed by well-trained registered dietitians, who calculated their nutrient intake from the dietary records and photographs. The dietary records were analyzed using nutrient analysis software (Excel Eiyokun Ver. 7.0; Kenpakusha, Tokyo, Japan). To avoid the participants based on energy intake (EI) misreporting, we used arbitrary cut-off points set at <500 kcal/day for under-reporting and >3500 kcal/day for over-reporting [35,36]. However, participants who reported implausibly high or low EI levels were not included in the study.

### 2.5. Inclusion and Exclusion from the Participants

We included 14 participants from the participants who had muscle weakness, 4 who had poor walking function, 9 who had poor cognitive function, 9 who had underweight, 22 who had overweight, 18 who had type 2 diabetes, 19 who had dyslipidemia, and 31 who had hypertension. Thirty-six of the included individuals overlapped. The total participants were 58. As described above, the detailed criteria are shown in the Methods section. For 58 participants, we excluded 2 participants from the analyses who missing data for triaxial accelerometer or dietary record.

### 2.6. Statistical Analysis

Data are expressed as means ± standard deviations (SDs), unless otherwise stated. Protein intake is reported as crude protein intake per kg body weight (g/kg BW). Initially, partial correlation controlling analyses were then used to ascertain if there were associations between %FFM and these variables, independent of age and sex. Subsequently, multiple linear regression analysis was used to explain the variation in %FFM according to the nutritional and physical activity parameters, after adjustment for age and sex. The residuals in the dependent variable were approximately normally distributed. Multicollinearity assumptions were also tested using tolerance and the variance inflation factor (VIF). In the present study, a tolerance of <0.10 or a VIF of >10 was considered to be indicative of multicollinearity. *P* < 0.05 was considered to represent statistical significance. Statistical analyses were performed using SPSS for Windows (version 23.0; IBM Inc., Armonk, NY, USA).

## 3. Results

Table 2 shows the relationships between %FFM and nutritional and physical activity parameters. %FFM was significantly negatively correlated with BW and BMI (BW, r = −0.321, *p* < 0.05; BMI, r = −0.604, *p* < 0.001). These negative correlations remained after adjustment for age and sex (BW, r = −0.719, *p* < 0.001; BMI, r = −0.753, *p* < 0.001). There was a significant positive correlation between %FFM and protein intake (r = 0.541, *p* < 0.001), which strengthened after adjustment for age and sex (r = 0.652, *p* < 0.001). There was also a significant positive correlation between %FFM and MVPA (r = 0.389, *p* < 0.01), which remained after adjustment for age and sex (r = 0.350, *p* < 0.01). There was no correlation between PAL and %FFM (r = 0.122, *p* = 0.372), but a significant correlation emerged after adjustment for age and sex (r = 0.345, *p* < 0.05). According to the multiple linear regression analysis (Table 3), age and sex together explained 22% of the variation in %FFM (Model 1). In multiple linear regression analysis, when PAL/MVPA or protein intake were included, 31%/32% and 55%, respectively, of the variation in %FFM was explained after adjustment for age and sex. Moreover, the addition of both PAL/MVPA and protein intake explained 61%/60%, respectively, of the variation in %FFM after adjustment for age and sex.

## 4. Discussion

The purpose of this cross-sectional study was to investigate the relationships between indices of physical activity, protein intake, and FFM, and to determine whether high PAL and protein intake might be associated with higher FFM in community-dwelling, older people with mild disability. This showed that %FFM, measured using the isotope dilution method, independently correlates with protein intake (g/kg), PAL, and MVPA. This is the first study, of which we are aware, to identify potential contributing factors to %FFM from physical activity indexes using PAL (doubly labelled water method) and a tri-axial accelerometer. The study found that a combination of physical activities (PAL or MVPA) and protein intake was positively related to fat-free mass among older adults with mild disability.

### 4.1. Levels of Physical Activity and Protein Intake by Participants

Several studies of geriatric populations in Western countries have been published that used DLW, but only a few have been conducted in older Asian or Japanese populations [26,37]. One recent study found that the mean ± SD daily step count was 8334 ± 3591 and PAL was 1.90 ± 0.29 in people with a mean age of 73 years who were not highly active [26]. The PAL for the older people in the present study was 1.77 ± 0.24 and the step count was 6199 ± 2747. The main reason for the lower step count in the present study compared with the previous study, also conducted in older Japanese participants, is that the participants in the present study have disease conditions that predisposed towards sarcopenia and frailty. The lower step count is likely to be at least partially responsible for the lower PAL in the present study [26].

Participants in the present study had a mean protein intake of 1.28 ± 0.32 g/kg/day, which is similar to that recorded for older Japanese people in one previous study [38], but higher than that measured in other studies [13,39,40,41,42]. Hernandez-Alonso et al. reported that higher animal protein consumption, particularly red meat, was associated with an increased risk of cardiovascular event and cardiovascular, cancer, and total death, compared with moderate consumption [40]. Our findings suggest that a high total protein intake is helpful in achieving high %FFM. However, we suggest that although total protein intake is clearly important, older people must also be careful about the source and type of protein consumed.

### 4.2. The Relationship between FFM and Physical Activity

The high PAL in the participants in the present study was associated with high FFM, likely because it promotes net muscle protein anabolism and leads to specific metabolic and morphological adaptations in skeletal muscle tissue [43]. Previous studies have evaluated the relationship between physical activity and FFM [44,45,46,47,48] and shown that LPA, MVPA, and the amount of leisure time are associated with FFM in older people [46,47,48]. Furthermore, Park et al. have reported that the skeletal muscle mass of older people shows a weak, but statistically significant, positive association with the duration of daily exercise conducted at an intensity of >3 METs [47]. Consistent with this, the present study identified a positive relationship between %FFM and MVPA after adjustment for age and sex.

The present study provides evidence that a high PAL protects against the loss of FFM, because PAL also correlated with %FFM. However, in contrast, Speakman and Westerterp found no relationship between PAL and FFM (kg) in a large cross-sectional study [45], as did another smaller study [49] of an older population. One of the reasons for this inconsistency may be differences in lifestyle between the countries in which the studies were conducted. The older Japanese population has a relatively high PAL [26,37] compared with the older population of other countries [45,50], and indeed the PAL measured in our older participants (1.77 ± 0.24) was similar to that of participants of ~50 years elsewhere [45]. This difference in PAL between countries is likely to be reflected in the %FFM. We have also shown that PAL and MVPA significantly correlate (*p* < 0.001, r = 0.508) (data not shown). Thus, an increase in MVPA in daily life in the elderly will lead to an increase in PAL. The results of the previous studies suggest that the intensity of daily activity, rather than its duration, may be important for the protection of FFM. There are various reasons why muscle mass is lost with age, but these results suggest that increasing the intensity of physical activity and overall PAL is effective at maintaining %FFM in older people with mild disability.

### 4.3. The Relationship between FFM and Protein Intake

Anabolic resistance, characterized by a reduction in anabolic signaling for protein synthesis, may be ameliorated by an increase in protein intake, which has been shown to stimulate muscle protein synthesis in older people [14,51]. Furthermore, previous studies have shown that a protein intake higher than the RDA is effective at maintaining FFM in older people [13,17,52]. For example, Houston et al. reported that for older adults who lost weight over the 3-y period, lower protein intake was associated with greater loss of lean mass [52], and Morris et al. [17] reported that higher protein intake is associated with higher appendicular skeletal muscle mass. The outcomes of both of these longitudinal studies suggest that benefits are accrued by participants who consume more than the RDA for protein of 0·8 g/kg/day. The findings of the present study are consistent with these previous findings, because higher protein intake was associated with higher %FFM. Thus, high protein intake may contribute to the maintenance of %FFM in older people with mild disability.

### 4.4. The Relationship between FFM and a Combination of Protein Intake and Physical Activity

A combination of exercise and higher protein intake seems to be the most effective strategy for the maintenance of FFM [16,17,53]. The present study (Table 3) has shown that a combination of both protein intake and PAL contribute substantially to %FFM after adjustment for age and sex than each variable alone. Indeed, Martone et al. stated in a review article that “the combination of exercise with increased protein intake seems to be the most plausible strategy to overcome such an issue” [53]. Morris et al. have also shown that muscle-strengthening combined with high protein intake is more efficient at increasing skeletal muscle mass than high protein intake alone [17], and Verreijen et al. found that a combination of a high-protein diet and a resistance exercise program increased FFM more effectively than diet or exercise alone during weight loss in older adults [54]. Thus, in summary, intervention studies have shown that FFM can be most effectively increased using a combination of exercise and protein intake. This conclusion is consistent with the results of the present cross-sectional study of community-based older people. Previous studies have also shown that exercise and/or an increase in general physical activity should be considered an important method of improving the anabolic response, which prevents a reduction in FFM and supports healthy aging [17,54,55]. Breen et al. have reported that short periods of relative muscle disuse lead to modest increases in markers of inflammation, a gradual reduction in insulin sensitivity, and blunting of feeding-induced muscle protein synthesis, all of which may transiently accelerate sarcopenia [55]. Therefore, a high %FFM in community-dwelling older people with mild disability may be maintained by encouraging a combination of high protein intake and high PAL.

### 4.5. Limitations

The present study had several limitations. First, it was cross-sectional, and therefore cause-and-effect and age-related effects cannot be inferred. A longitudinal study should be able to evaluate this possibility. Second, FFM hydration was assumed to be equal in all the participants (0·732) [56]; therefore, there may have been errors in the estimation of FM using FFM, associated with differences in adiposity and sex [57]. Third, PAL was estimated without directly measuring RMR. Miyake et al. have reported that the RMR equation used in this study is accurate for Japanese people [58]. Finally, the self-reported dietary records used to estimate protein intake may be susceptible to inaccurate reporting. We attempted to minimize this potential error by asking the participants to take photographs of the meals they ate.

## 5. Conclusions

In summary, our findings of the present cross-sectional, population-based study show that consumption of at least the current RDA for protein and high PAL are beneficial for the maintenance of FFM in older people with mild disability, especially when used in combination.

## Figures and Tables

**Table 1:** Participants’ characteristics.

Parameter	All *n* = 56		Men *n* = 17		Women *n* = 39	
	Mean	SD	Mean	SD	Mean	SD
Age, years	71.8	6.9	71.1	6.6	72.1	6.9
Height, cm	154	9	162.9	6.3	149.4	5.6
Body weight, kg	54.5	12.2	63.8	12.3	50.5	9.4
Body mass index, kg/m^2^	23.0	3.8	23.9	3.3	22.6	3.9
Fat-free mass, kg	36.9	8.2	46.2	7.7	32.8	3.8
Fat-free mass, %	68.1	7.5	73.0	7.3	65.9	6.4
Energy intake, kcal/day	1814	327	2066	349	1704	240
Energy expenditure, kcal/day	1853	375	2126	440	1734	260
Energy expenditure, kcal/kg/day	34.5	5.3	33.5	4.5	34.9	5.5
**Nutritional parameter**						
Protein intake, g/day	71.5	14.0	79.9	13.4	67.9	12.3
Protein intake, g/kg BW/day	1.28	0.32	1.25	0.28	1.29	0.33
Protein intake, g/kg FFM/day	1.99	0.40	1.75	0.29	2.09	0.40
Fat intake, g/day	57.4	15.6	62.6	13.1	55.1	15.8
Carbohydrate intake, g/day	239	41	271	40	225	32
**Protein source**						
Soy protein, g/day	9.4	7.9	9.6	6.1	9.3	8.5
Fish and shellfish protein, g/day	12.4	6.9	14.0	5.3	11.7	6.4
Meat protein, g/day	12.2	6.9	14.4	7.3	11.2	6.4
Egg protein, g/day	4.8	3.0	5.5	2.9	4.5	2.9
Milk protein, g/day	19.5	14.5	21.1	17.8	18.8	12.5
Other protein, g/day	13.2	12.3	15.3	11.7	12.4	12.3
**Distribution of protein intake across eating occasions**						
Breakfast protein, g/meal	16.5	6.2	17.5	6.7	16.0	5.8
Lunch protein, g/meal	21.3	6.6	22.2	7.2	21.0	6.2
Supper protein, g/meal	30.0	7.7	34.5	6.9	28.0	7.1
Snack protein, g/meal	3.8	5.5	5.8	6.7	2.9	4.5
**Physical activity parameter**						
PAL	1.77	0.24	1.62	0.20	1.84	0.22
Steps, counts/day	6199	2747	6535	2903	6053	2626
Sedentary, min/day	200	46	167	39	214	40
LPA, min/day	332	87	259	53	364	78
MVPA, min/day	30	20	32	22	28	19

BW: body weight, FFM: fat-free mass, PAL: physical activity level, LPA: light physical activity, MVPA: moderate-to-vigorous-physical activity

**Table 2:** Associations between the percentage of FFM and other parameters.

	Crude Values		Values Adjusted for Age and Sex	
Parameter	r	*p*	r	*p*
**Physical parameter**				
Age, years	−0.198	0.144		
Height, cm	0.236	0.080	−0.191	0.167
Body weight, kg	−0.321	0.016	−0.719	<0.001
BMI, kg/m^2^	−0.604	<0.001	−0.753	<0.001
**Nutritional parameter**				
Energy intake, kcal/day	0.207	0.126	-0.054	0.700
Protein intake, g/day	0.295	0.027	0.142	0.305
Protein intake, g/kg/day	0.541	<0.001	0.652	<0.001
Fat intake, g/day	−0.026	0.853	−0.192	0.163
Carbohydrate intake, g/day	0.215	0.111	−0.042	0.765
**Physical activity parameter**				
PAL	0.122	0.372	0.345	0.011
Steps, counts/day	0.234	0.083	0.161	0.246
Sedentary, min/day	−0.163	0.231	0.076	0.586
LPA, min/day	−0.117	0.403	0.137	0.321
MVPA, min/day	0.389	0.003	0.350	0.009

BMI: body mass index, PAL: physical activity level, LPA: light physical activity, MVPA: moderate-to-vigorous-physical activity.

**Table 3:** Multiple linear regression analysis of the relationship of %FFM with PAL and protein intake.

	R^2^	*p* Value	Included Independent Variables	Standardized Coefficient (β)	*p* Value
Model 1	0.221	<0.001	Age	−0.167	0.176
			Sex	−0.428	0.001
Model 2	0.314	<0.001	Age	−0.059	0.632
			Sex	−0.588	<0.001
			PAL	0.357	0.011
Model 3	0.317	<0.001	Age	−0.020	0.879
			Sex	−0.407	<0.001
			MVPA	0.344	0.009
Model 4	0.552	<0.001	Age	−0.175	0.066
			Sex	−0.467	<0.001
			Protein intake	0.576	<0.001
Model 5	0.611	<0.001	Age	−0.088	0.348
			Sex	−0.593	<0.001
			PAL	0.287	0.008
			Protein intake	0.550	<0.001
Model 6	0.599	<0.001	Age	−0.070	0.485
			Sex	−0.450	< 0.001
			MVPA	0.245	0.018
			Protein intake	0.540	< 0.001

R^2^: total variance explained by the model. PAL: physical activity level, MVPA: moderate-to-vigorous physical activity.
